# Marine Heatwave Event Maps in the Baltic Sea (1982–2023): A Gridded Dataset from Satellite-Derived L4 SST

**DOI:** 10.1038/s41597-025-06251-7

**Published:** 2025-12-10

**Authors:** Behzad Bashiri, Amirhossein Barzandeh, Aarne Männik, Rivo Uiboupin, Urmas Raudsepp

**Affiliations:** Department of Marine Systems, Tallinn University of Technology, Tallinn, 12618 Estonia

**Keywords:** Physical oceanography, Natural hazards

## Abstract

Marine heatwaves (MHWs) are critical to monitor as they can cause widespread impacts on marine ecosystems, fisheries, and coastal communities. This manuscript presents a high-resolution dataset documenting MHW events in the Baltic Sea over a 42-year period (1982–2023), based on daily satellite reprocessed observations at 0.02° × 0.02° spatial resolution, including the three-dimensional (longitude × latitude × time) array of daily MHW intensity values, which captures the spatial and temporal evolution of each event across the Baltic Sea. Given the Baltic Sea’s heightened sensitivity to rapid warming and climate change, this resource provides valuable insights into regional oceanographic extremes. The dataset supports detailed analyses of MHW frequency, intensity, and duration, aiding research in climate science, marine ecology, and coastal management. By offering long-term, event-based records, it enables investigations into thermal stress impacts on ecosystems and the evaluation of regional climate model performance. This open-access dataset is designed to foster interdisciplinary studies addressing the environmental and societal challenges associated with ongoing warming in the Baltic Sea.

## Background & Summary

Prolonged episodes of anomalously high sea surface temperatures (SST) are known as marine heatwaves (MHWs)^1,2^. These events can have profound impacts on marine ecosystems^3,4^ and are strongly influenced by large-scale climate modes and processes^5,6^. While global MHW analyses provide a general view of warming patterns and their implications, more regional studies are needed to understand localized dynamics^7,8^.

The Baltic Sea, a high-latitude semi-enclosed basin, is uniquely vulnerable to climate change due to its brackish water, shallow depth, strong stratification, and limited exchange with the open ocean^9,10^. Warming at a rate faster than the global average, the Baltic is experiencing more frequent and intense MHWs^11,12^, which consolidate existing challenges such as eutrophication, hypoxia, and biodiversity loss^13,14^. Unlike global analyses, regional studies in the Baltic Sea capture the nuanced interactions of warming, stratification, and human activities, providing crucial insights for tailored adaptation strategies^15^. Global MHW analyses, while valuable for identifying large-scale patterns, often operate at spatial resolutions too coarse to resolve the complex coastline and bathymetry of regional seas^16–18^. Therefore, long-term datasets for specific basins are essential because the consequences of MHWs in such environments often differ significantly from those observed in large open-ocean systems. Without basin-specific data, the unique dynamics of these environments may be overshadowed by global averages, leading to the misinterpretation of the severity and nuances of their effects^19^. As one of the world’s most sensitive marine environments^20–22^, the Baltic’s unique characteristics amplify its susceptibility to warming, making comprehensive datasets indispensable for predicting future trends and mitigating impacts effectively^23,24^.

Understanding when and where MHWs occur—and how their intensity and duration evolve—is critical for linking physical extremes to ecological responses in the Baltic Sea. Regional datasets enable researchers to resolve how MHWs interact with local Baltic Sea processes such as eutrophication^25^, nutrient cycling^26^, and species distributions^27,28^, thereby strengthening the attribution of observed biological changes to specific thermal events. These datasets also inform ecosystem-based management by identifying spatial hotspots of recurrent exposure and temporal windows of elevated risk. High-resolution, long-term observations are further indispensable for evaluating and refining global and regional climate models in a semi-enclosed basin, thereby increasing confidence in projections of future warming. With accelerating advancements in data-driven research and applied artificial intelligence^29–31^, such datasets also open opportunities for predictive modeling and early-warning systems. Nevertheless, their greatest value lies in advancing process-based understanding and supporting adaptive management strategies tailored to the Baltic Sea’s unique sensitivities. This study introduces a long-term, high-resolution dataset of MHWs in the Baltic Sea. The dataset has been derived from reprocessed SST observations provided by the Copernicus Marine Service, specifically the SST-BAL-SST-L4-REP-OBSERVATIONS-010-016 product^32,33^. This multi-sensor, Level 4 optimally interpolated product is based on infrared satellite observations. Spanning 42 years (1982–2023), the dataset provides daily reprocessed observations at a fine spatial resolution of 0.02° × 0.02°. Building on the comprehensive analysis and evaluation by Bashiri *et al*.^34^ this paper aims to formally introduce and share the dataset to facilitate its application in further research across various scientific and operational domains.

## Methods

The detailed methodology is available in Bashiri *et al*.^34^. In summary, the identification of MHWs from the derived SST data follows the framework established by Hobday *et al*.^35^ and is implemented using a MATLAB toolbox^36,37^. Particularly, our MHW detection relies on SST surpassing the 90th percentile of the baseline period for a minimum of five consecutive days at each data grid. We have considered that the use of a time-fixed baseline for identifying marine heatwaves could be affected by long-term climate change and modulated by certain global climatic phenomena^38–40^. Prior studies have underscored the substantial relationship between the atmospheric and oceanic conditions of the Baltic region and fluctuations in two principal climatic indicators: North Atlantic Oscillation (NAO) and Atlantic Multidecadal Oscillation (AMO)^41–43^. To ensure a robust baseline for detecting MHWs in the Baltic Sea, a neutral climatology was established using a minimum 10-year time window, selecting the period 1992–2002 based on the lowest average absolute values of NAO and AMO indices. Eventually, all MHWs within the study period were identified, and four key characteristics—intensity, duration, area, and a composite index that integrates these features to enable comparison between different MHW events—were extracted^34^. The gridded SST fields allowed MHW detection at each grid point, with intensity defined as the difference between the observed SST and the corresponding daily climatological baseline (whenever SST exceeded the 90th percentile of baseline values for a minimum of five consecutive days). A distinct MHW event was considered terminated when no MHW conditions were detected at any grid point across the entire Baltic Sea domain for at least one day; any subsequent exceedance was then classified as the start of a new event.

## Data Record

We identified 189 distinct MHW events in the Baltic Sea between 1982 and 2023. For each MHW event, we generated a separate NetCDF file containing the variables listed in Table 1. These files provide detailed data for each event, ensuring easy accessibility and usability for further analysis, and are freely available at the Science Data Bank^44^ (10.57760/sciencedb.22947).

**Table 1:** Content of each MHW data file.

Variable name	Unit	Dimension	Description
longitude	°E	x-axis (1051)	Longitudes of grid points in degrees East, ranging from 9.00° to 30.00° with an increment of 0.02°.
latitude	°N	y-axis (626)	Latitudes of grid points in degrees North, ranging from 53.50° to 66.00° with an increment of 0.02°.
time	seconds since 1970-01-01	time (event duration in days)	Time axis representing daily data, where the number of time steps equals the duration of each MHW event
MHW_int_maps	kelvin	longitude × latitude × time	3D array of daily MHW intensity values. A value of 0 indicates no MHW at a given location and time; NaN values represent land.
MHW_int_ts	kelvin	time	Time series of the daily mean intensity of the MHW event over the affected Baltic Sea area.
MHW_area_ts	km^2^	time	Time series of the daily total area affected by the MHW over the Baltic Sea.

In addition to the primary set of 189 NetCDF files, a supplementary data file is provided to enhance the usability of the dataset^44^. This file includes climatology maps (Variable name: **mclim**), stored as a 3D numeric matrix with dimensions of 1051 (longitude), 626 (latitude), and 366 (Julian days), with units in kelvin. These maps represent the daily climatology for each grid point across the year and serve as the baseline for marine heatwave (MHW) detection. The file also contains MHW detection thresholds (Variable name: **m90**), another 3D numeric matrix with the same dimensions and units, which holds the 90th percentile threshold values used to identify MHWs at each grid point for every Julian day. Additionally, the grid cell area (Variable name: **BALarea**) is provided as a 2D numeric matrix with dimensions of 1051 (longitude) and 626 (latitude), with units in square kilometers (km²), indicating the physical area of each grid cell. Together, these supplementary components provide essential reference data for the detection and analysis of MHW events, supporting a broad range of scientific and operational applications.

## Technical Validation

Conceptually, the quality of the MHW event dataset^44^ was ensured through the careful selection of a climatological baseline and the rigorous application of established detection methods. The use of a fixed historical baseline — rather than a moving or recent climatology — was a deliberate choice to enable consistent long-term comparisons. However, to ensure that the selected baseline remains representative under a changing climate, we verified its neutrality through a documented NAO/AMO analysis^34^. This approach mitigates the risk of biasing MHW detection either toward an overly warm or cool reference state. In addition, MHW events were detected using the internationally recognized methodology of Hobday *et al*.^35^. The 90th-percentile threshold ensures that the detection criteria are neither too permissive (avoiding the misclassification of ordinary warm periods) nor overly stringent (capturing all significant heat anomalies) across the full 1982–2023 analysis window. All MHW detection procedures were performed using the m_mhw MATLAB toolbox^36^, which has been previously validated and widely adopted by users and researchers. The reliability of the dataset is further supported by the quality of the underlying SST input data^45^. We used the Baltic Sea SST Reprocessed product, which has undergone extensive calibration and validation by the Danish Meteorological Institute and the Copernicus Marine Environment Monitoring Service (CMEMS). Detailed validation processes for the SST product are available in its Quality Information Document (QUID)^46^. In addition to the inherent quality controls, we further refined the initial satellite data by selectively cleaning it to focus exclusively on the Baltic Sea domain. Non-relevant water bodies (e.g., small inland lakes) and spatial noise were systematically masked using an external bathymetry file^47^ derived from the CMEMS Baltic Sea Reanalysis supplementary data. To address spatial inconsistencies between the two datasets, the bathymetry-derived mask was interpolated onto the SST data grid using a nearest-neighbor method. It is important to note that our technical validation efforts prioritized methodological consistency and climatological reasoning during data generation.

Once the dataset was finalized, we validated it using moored *in situ* water temperature data from the CMEMS product *Baltic Sea – In Situ Near Real-Time Observations*^48^. These observations are provided at sub-daily resolution (typically every 30–60 minutes) and represent direct measurements of near-surface water temperature. Each record includes a quality-control flag (QCF) ranging from 0 to 9. We retained only measurements flagged as *good_data* (QCF = 1) or *probably_good_data* (QCF = 2), excluding all others—including *no_qc_performed*, *bad*, or manipulated data—and then calculated daily means from the remaining records. Because mooring sensors are typically positioned at depths of 0.5–1.0 m, we considered them representative of SST, using the 0.5 m measurement where available, otherwise the 1.0 m record.

To enable direct comparison with our gridded dataset, we applied the same daily climatological baseline (variable **mclim**) and 90th-percentile threshold (variable **m90**) provided in our *BALMHW_supp.nc*^44^, which were used for our MHW detection. For each *in situ* station, we extracted the gridded climatology and threshold from the nearest grid point (i, j) to the station’s location. Daily temperature anomalies were then calculated as:

$$\triangle {{SST}}_{{in\; situ}}(t)={{SST}}_{{in\; situ}}(t)-m90(i,j,{t}_{c})$$

where $$T$$ is each day of the *in situ* record, and $${t}_{c}$$ is the corresponding climatological calendar day. To follow the core approach of MHW detection—where events are defined relative to the 90^th^ percentile—negative anomalies were treated as non-events (NaN). It should be noted that the commonly applied minor criterion of at least five consecutive days above the threshold was not enforced, as the objective here was to validate the agreement between datasets on a day-to-day basis rather than to identify discrete events. To assess the agreement in MHW occurrence between the observations and our dataset^44^, we compared, for each day and station, the binary MHW classification derived from *in situ* anomalies with the corresponding (or nearest) grid-cell values from the MHW event files (MHW_int_maps(i,j,t) in BALMHW_$event_number$.nc). Agreement was defined when both datasets either indicated an MHW (positive anomaly) or no event (NaN). Disagreement occurred only when one dataset detected an event while the other did not.

To quantify this, we calculated the Percentage Agreement (PA):

$${PA}\left( \% \right)=\frac{{Number\; of\; days\; in\; agreement}}{T}\times 100$$

Where $T$ is the total number of days considered. ${PA}$ thus provides a categorical measure of event detection consistency. To further assess MHW intensity consistency, we calculated *in situ* event intensities for all days where both datasets indicated an MHW, using the same daily climatology applied in our dataset^44^:

$${{MHW}}_{\mathrm{int},{in\; situ}}(t)={{SST}}_{{in\; situ}}(t)-{mclim}(i,j,{t}_{c})$$

and then computed the Mean Absolute Error (MAE):

$${{MAE}}_{{in\; situ}}=\frac{\mathop{\sum }\limits_{t=1}^{{T}_{{agreement}}}\left|{{MHW}}_{\mathrm{int},{in\; situ}}(t)-{\rm{MHW\_}}\mathrm{int}{\rm{\_maps}}(i,j,t)\right|}{{T}_{{agreement}}}$$

where ${T}_{{agreement}}$ is the number of days when both datasets indicate an event. MAE provides a quantitative measure of the intensity difference between *in situ* and gridded MHW estimates.

**Fig. 1:**
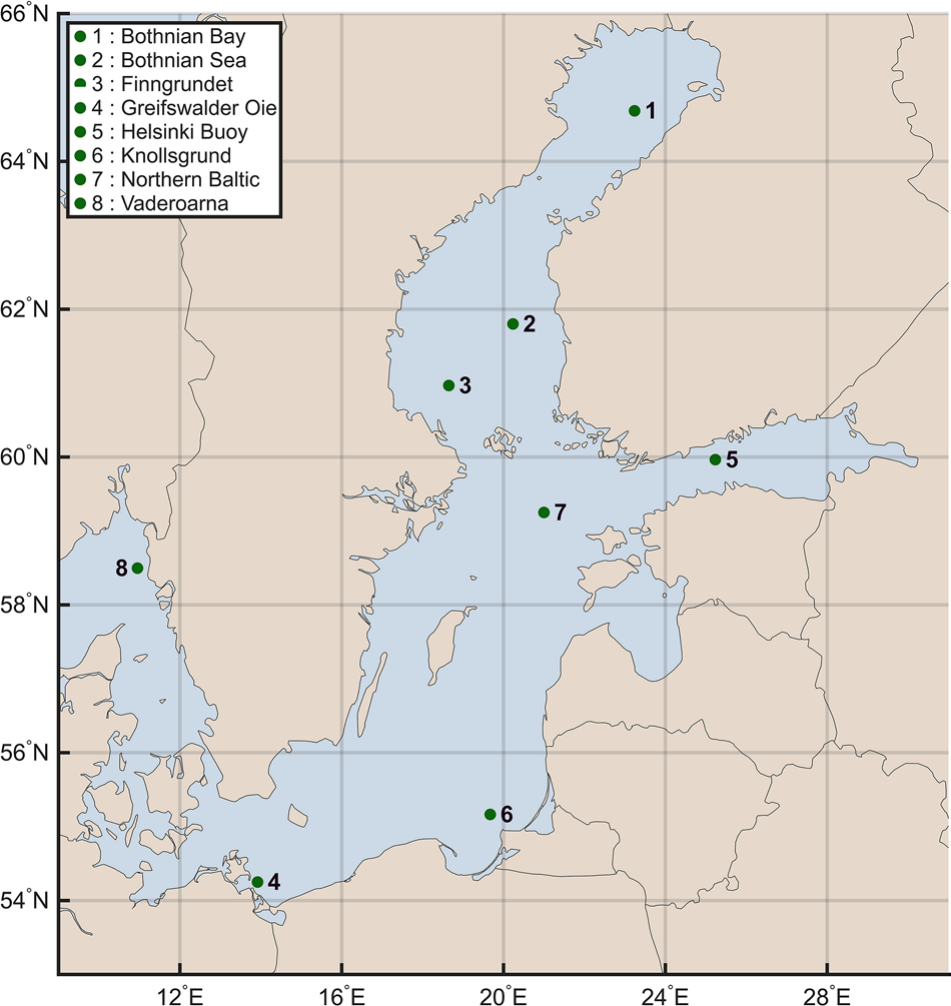
Station of the moored *in situ* water temperature data used for validation in the present study.

For benchmarking, we selected two relatively long-duration events (event numbers 183 and 187 in the metadata) and identified eight mooring stations with sufficient high-quality data for both events (QCF = 1 or 2). According to the provided metadata (BALMHW_data_info.csv), event 183 spans 5 August 2020–19 August 2021 and event 187 spans 1 August 2022–25 July 2023, with each lasting nearly one year. These extended durations allow for more comprehensive benchmarking, and their occurrence in recent years coincides with a higher proportion of observational data meeting QCF 1 or 2 standards. Furthermore, Bashiri *et al*.^34^ reported that the highest area-averaged MHW intensities have occurred in recent years, particularly in 2020, increasing the likelihood that our dataset contains a greater number of available grid points for MHW intensity values and thereby enabling more robust validation. For each station (Fig. 1), we calculated PA and MAE as described above, allowing event-specific validation of both MHW occurrence and intensity in the dataset^44^.

**Fig. 2:**
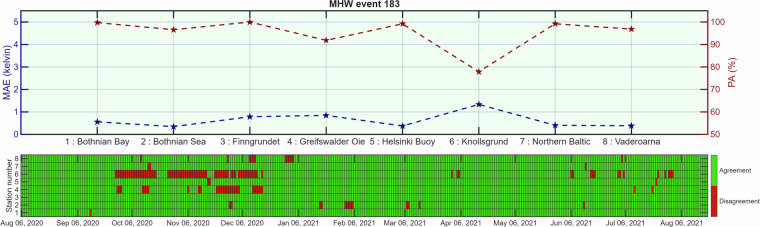
Comparison of MHW event 183 between the Baltic Sea MHW dataset^44^ and eight moored *in situ* stations^48^.

**Fig. 3:**
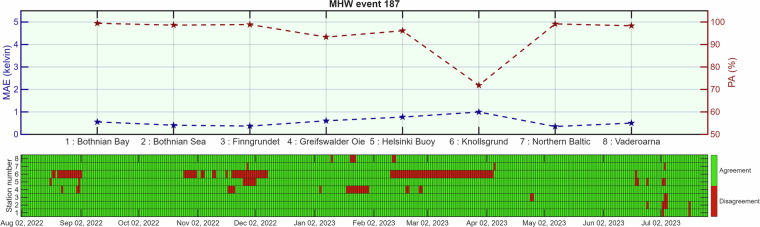
Comparison of MHW event 187 between the Baltic Sea MHW dataset^44^ and eight moored *in situ* stations^48^.

For both events 183 and 187 (Figs. 2, 3), agreement between *in situ* and gridded data was consistently high (>90%) across most stations. Figures 2, 3 present a station-by-station comparison: the top panels show the MAE, (blue axis) of MHW intensity and the PA, (red axis) between the Baltic Sea MHW datasetand *in situ* observations, while the bottom panels display daily agreement (green) and disagreement (red) between MHW detection in the gridded product and *in situ* time series for stations 1–8 (listed by name). Station 6 (Knollsgrund) showed slightly lower agreement, with short periods of mismatch during late autumn and early winter in event 183 and somewhat more frequent mismatches during early autumn in event 187. Nevertheless, the MAE in intensity remained low (≈1 K) for all stations and both events, indicating that mismatches occurred close to the detection threshold rather than reflecting large intensity discrepancies. Taken together, the high PA values and low MAEs across geographically diverse stations and multiple seasons demonstrate that the dataset reliably captures both the occurrence and intensity of MHWs.

## Data Overview

**Fig. 4:**
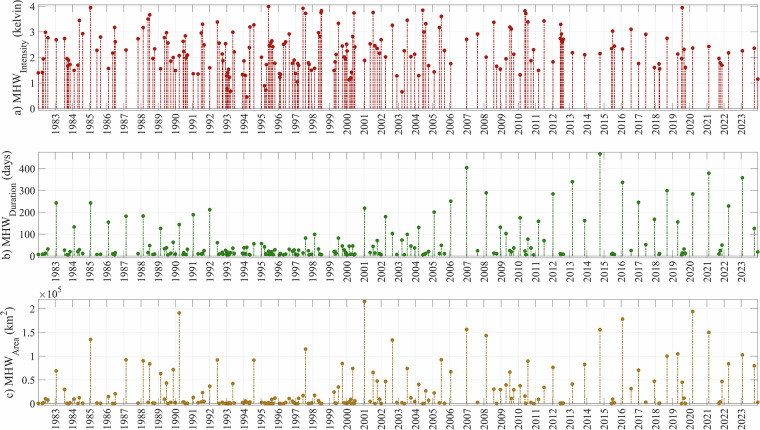
Temporal distribution of mean characteristics of MHW events record in the Baltic Sea 1982–2023.

Figure 4 offers a comprehensive overview of all MHW events during the period 1982–2023, derived from the generated MHW data files^44^. The figure is structured into subplots, each presenting the temporal distribution of key event characteristics, including average intensity, spatial extent, and duration, providing insights into the dynamics and trends of MHW events over the analyzed period. Each bar in the subplots is centered on the central date of the respective MHW event. For the duration values, a bar with a height of A at time T represents A/2 days prior to the event start and A/2 days after the event ended. Similarly, the mean area and intensity values are aligned to the same central time vector, ensuring consistency in the temporal representation of the data. This visualization highlights the temporal distribution and characteristics of MHW events, offering valuable insights into their dynamics over the analyzed period.

Between 1982 and 2023, a total of 189 distinct MHW events were identified in the Baltic Sea, and their data are presented and described in this paper. The median duration of these events is 18 days, meaning that half of the events lasted 18 days or less. The 80th percentile of event duration is 98 days, indicating that the majority of events lasted fewer than 14 weeks. To facilitate comparison, events were categorized by duration: short events (<18 days), medium events (18–98 days), and long events (>98 days).

Table 2 summarizes descriptive statistics for each category, including the maximum intensity and maximum area. These values can be computed from the event files by identifying the maximum index in the time-series variables **MHW_int_ts** and **MHW_area_ts** listed in Table 1. To further characterize data evolution, events were grouped by the timing of their maximum intensity and maximum area relative to their start date: the first third (0–33%), middle third (34–66%), or final third (67–100%) of the event’s duration.

**Table 2:** Descriptive statistics of MHW maximum intensity and area in the Baltic Sea, grouped by event duration and timing of peaks relative to the event start.

MHW events	Max intensity			Max area		
	1^st^ third	2^nd^ third	3^rd^ third	1^st^ third	2^nd^ third	3^rd^ third
MHW_duration_ < 18 days	17%	36%	47%	40%	56%	4%
18 ≤ MHW_duration_ ≤ 98 days	36%	38%	26%	16%	55%	29%
MHW_duration_ > 98 days	32%	13%	55%	20%	48%	32%

Overall, Table 2 underscores the diverse and variable behavior of MHW events in the Baltic Sea, highlighting the need for more in-depth analysis to unravel their underlying dynamics. Despite the inherent variability, even this preliminary level of analysis reveals some consistent patterns. For example, the majority of long-term events tend to reach their maximum intensity after two-thirds of their duration has passed. While this constitutes a relatively robust finding within the broader dataset, the wide-ranging diversity of event characteristics suggests that significant complexities remain unexplored. Further investigation could uncover additional insights by employing advanced analytical techniques, such as clustering events based on distinct traits like duration, spatial extent, or intensity trends. Differentiating between past and recent events could also provide a clearer understanding of temporal shifts in MHW behavior. Such investigations would allow for a more in-depth understanding of these occurrences, hence facilitating predictive modeling and tailored management techniques.

The gridded MHW data provided by this study can be used as a resource for interdisciplinary research in the future, bridging data science, climatology, and oceanography. It makes it possible to apply state-of-the-art AI techniques that have the potential to be revolutionary, opening the door for creative answers to the problems these extraordinary occurrences in a warming planet present.

## Data Availability

The dataset of 189 distinct marine MHW events in the Baltic Sea (1982–2023), including event-based NetCDF files, supplementary reference data, and metadata, is publicly available at the *Science Data Bank*: 10.57760/sciencedb.22947. Each NetCDF file contains longitude, latitude, time, daily MHW intensity maps (*MHW_int_maps*), daily mean intensity time series (*MHW_int_ts*), and daily affected area time series (*MHW_area_ts*). The supplementary file (**BALMHW_supp.nc**) provides (i) climatology maps (*mclim*), (ii) detection thresholds (*m90*), and (iii) grid cell areas (*BALarea*), stored as a 2D matrix (longitude × latitude). The *BALarea* variable represents the physical area of each grid cell in km², calculated by converting geographic coordinates (longitude, latitude in degrees; WGS84/EPSG:4326). The metadata file (**BALMHW_data_info.csv**) contains event-specific information, including identifiers, durations, and temporal coverage^44^. The underlying sea surface temperature (SST) input product is the *Baltic Sea SST Reprocessed dataset*^45^ from E.U. Copernicus Marine Service Information (CMEMS), Marine Data Store (MDS), available at: 10.48670/moi-00156. Validation was performed using *Baltic Sea- In Situ Near Real Time Observations*^48^ - moored *in situ* water temperature observations from E.U. CMEMS, MDS, available at: 10.48670/moi-00032. Only measurements flagged as good or probably good (QCF = 1 or 2) were retained for the analysis. All datasets used in the study and produced with this study are openly accessible without restrictions, and repository metadata provides full guidance on file structure and usage^44–48^.

## Code availability

The source code packages for reproducing the data presented in this paper, as well as the sample result plotting scripts, are available for download at the following link: 10.5281/zenodo.17207403.
